# Adipose Tissue Immunometabolism and Apoptotic Cell Clearance

**DOI:** 10.3390/cells10092288

**Published:** 2021-09-02

**Authors:** Tamás Röszer

**Affiliations:** Institute of Neurobiology, Ulm University, Albert-Einstein-Allee 1, 89081 Ulm, Germany; tamas.roeszer@uni-ulm.de; Tel.: +49-(0)7-3150-22629

**Keywords:** apoptosis, obesity, meta-inflammation, immunometabolism, macrophage, M2 macrophage, efferocytosis, phagocytosis

## Abstract

The safe removal of apoptotic debris by macrophages—often referred to as efferocytosis—is crucial for maintaining tissue integrity and preventing self-immunity or tissue damaging inflammation. Macrophages clear tissues of hazardous materials from dying cells and ultimately adopt a pro-resolving activation state. However, adipocyte apoptosis is an inflammation-generating process, and the removal of apoptotic adipocytes by so-called adipose tissue macrophages triggers a sequence of events that lead to meta-inflammation and obesity-associated metabolic diseases. Signals that allow apoptotic cells to control macrophage immune functions are complex and involve metabolites released by the apoptotic cells and also metabolites produced by the macrophages during the digestion of apoptotic cell contents. This review provides a concise summary of the adipocyte-derived metabolites that potentially control adipose tissue macrophage immune functions and, hence, may induce or alleviate adipose tissue inflammation.

## 1. Introduction: The Impact of Apoptotic Cell Clearance in Fat Depots

Apoptosis is an inevitable process in tissue development, and is a key component of the necessary physiological tissue turnover for proper tissue healing (reviewed in [1]). During this process, however, apoptotic cells pollute their surroundings with damage-associated molecular patterns, potential autoantigens, modified lipids and aged molecules, which can all impair tissue integrity and initiate immune responses. The safe removal of apoptotic debris is, hence, crucial to prevent self-immunity or the development of tissue damaging inflammation. This important task is performed by tissue-resident macrophages, which clear tissues of hazardous materials from the dead or dying cells without evoking inflammation [2]. The “immunologically silent” removal of apoptotic cell contents is particularly relevant when apoptotic cells are cleared from damaged, infected or inflamed tissues. Macrophages exposed to the content of apoptotic cells adopt a pro-resolving, anti-inflammatory or tolerogenic phenotype, which is often termed alternative- or M2-macrophage activation (reviewed in [3,4]). Apoptotic cell clearance induces interleukin-10 (IL-10) and tumor growth factor beta (TGFβ) production in macrophages concomitant with the expression of various M2-activation-associated molecules such as the high affinity scavenger receptor for the hemoglobin-haptoglobin complex CD163 and the mannose receptor CD206 [5].

Adipose tissue is unique in terms of its immune effects on apoptotic cell clearance, as adipocyte apoptosis triggers inflammatory cytokine responses in macrophages, and is a potential inducer of adipose tissue inflammation [6]. Classically, there are two types of adipose tissue in mammals: white adipose tissue (WAT) and brown adipose tissue (BAT) [7,8]. Adipocytes of the WAT accumulate neutral lipids in a large droplet [9], whereas BAT adipocytes have multilocular lipid droplets and high lipolytic activity, and oxidize fatty acids into ATP, and generate heat [10,11]. Beyond their role in fat metabolism, WAT and BAT have their own, specific, endocrine and immune functions [9]. Excess development of WAT leads to obesity, which is associated with a decline in BAT mass. Obesity is often related to meta-inflammation of the adipose tissue, which impairs insulin sensitivity in metabolic organs and triggers a decline in insulin production in the endocrine pancreas, ultimately manifesting as systemic insulin resistance, diabetes and metabolic syndrome [12]. With the rapidly increasing global incidence of obesity and obesity-associated diseases, investigating the mechanisms that control adipose tissue meta-inflammation is both timely and relevant.

## 2. Adipocyte Apoptosis Ignites Inflammation

**Figure 1:**
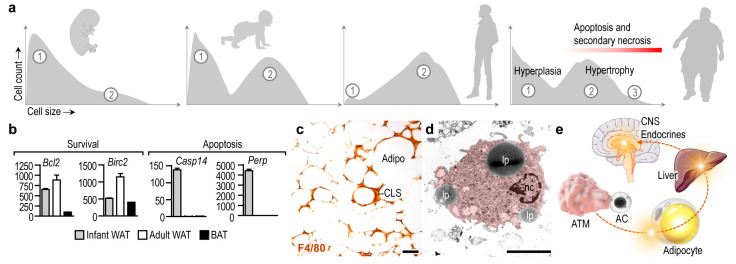
Adipocyte apoptosis and its metabolic impact. (**a**) In postnatal life, WAT undergoes differentiation and expansion. Differentiating adipocytes (cell population ①) are abundant after birth. In infancy, the prevalence of mature adipocytes (cell population ②) increases and, in adulthood, the lipid-storing mature adipocytes build-up the WAT. The number of differentiating and hypertrophic adipocytes (cell population ③) increases dramatically in obesity. Adipocyte hypertrophy is associated with apoptosis and secondary necrosis. Graphs modified from [20,22]. (**b**) Transcript level of survival factors *Bcl2* (BCL2 apoptosis regulator) and *Birc2* (baculoviral IAP repeat-containing 2) in infant and adult WAT and in adult BAT in the mouse. Note the high expression of the survival factors in adult WAT. In turn, the apoptosis markers *Casp14* (caspase 14) and *Perp* (TP53 apoptosis effector) are highly expressed in infant WAT, likely due to differentiation or remodeling of the WAT. Secondary analysis of next-generation sequencing experiments from the study [23]. (**c**) Immunohistochemical labeling of the macrophage marker F4/80 antigen in obese WAT in the mouse. Sample from the study [22]. CLS—crown like structure, Adipo—adipocyte, scale 50 μm. (**d**) Transmission electron microscopy of a mouse adipose tissue macrophage (ATM). Note the ingested lipid droplets (lp) that have been released by adipocytes. nc—nucleus, scale 2.5 μm. (**e**) Scheme summarizing the potential effect of ATMs on systemic metabolism. In response to apoptotic cells (AC), ATMs release mediators that impact the metabolism of adipocytes and other metabolic organs such as the liver. The resulting metabolic changes affect the central control of energy balance, by targeting endocrine organs and the central nervous system (CNS).

Number of adipocytes, which build up the adipose tissues, is stable over a prolonged period of time, and the mature adipocytes are relatively resistant to apoptosis [13,14,15]. Albeit adipocyte apoptosis appears during development and remodeling of the adipose tissue, and the adipokine C1q/TNF-related protein 9 (CTRP9) increases the uptake of apoptotic cells by macrophages [16], adipocyte number in the WAT depots is relatively constant during infancy and childhood [17]. The lipid content of the WAT increases along postnatal development due to the expansion of the cell volume of adipocytes [18], and a notable increase of adipocyte number appears at the onset of puberty [17]. In infancy, adipocytes undergo differentiation (Figure 1a) and activate lipolysis in response to insulin and β-adrenergic stimulation [19,20]. Two adipocyte populations can be identified later, in puberty and in adulthood: small, still maturing cells, and larger, lipid-laden mature adipocytes (Figure 1a) [18,21]. The number of mature adipocytes increases in severe obesity, and newly generated adipocytes also appear. An increase in adipocyte number is termed adipocyte hyperplasia, whereas an increment in cell volume due to lipid accumulation is termed adipocyte hypertrophy [14] (Figure 1a).

Adipocyte differentiation is associated with an increase in the expression of survival factors, such as the BCL2 apoptosis regulator and the baculoviral inhibitor of apoptosis repeat-containing 2 [13] (Figure 1b). WAT adipocytes are more resistant to apoptosis than BAT adipocytes [15]. Adipocyte apoptosis becomes prevalent under pathological conditions—for instance, lipodystrophy is associated with adipocyte apoptosis and necrosis [24]. Furthermore, the antiviral cytokine interferon alpha (IFNα) triggers adipocyte apoptosis both in vitro and in vivo in mice [25], and patients treated with HIV-1 protease inhibitors develop lipodystrophy due to adipocyte apoptosis [26]. In congenital generalized lipodystrophy, both WAT and BAT mass is lost due to adipocyte apoptosis and necrosis [24].

Hypertrophic adipocytes undergo apoptosis or secondary necrosis in obese adipose tissue (Figure 1a). Obesity is associated with meta-inflammation, which is a possible trigger of adipocyte death. TNFα, which is abundantly expressed in obese adipose tissue [12,27], induces both white and brown adipocyte apoptosis [15,28]. Human abdominal subcutaneous preadipocytes are more resistant to TNFα-induced apoptosis than are omental preadipocytes, and the distinct WAT depots contain at least two different adipocyte populations based on their resistance to TNFα-induced apoptosis [29]. Lipid overload in hypertrophic adipocytes can lead to the “spillover” of lipids into the cytosol, leading to so-called lipotoxicity and, ultimately, apoptosis [30]. Failure of fatty acid oxidation and oxidative phosphorylation initiates the mitochondrial pathway of apoptosis in adipocytes [31], and also triggers inflammatory cell death, termed pyroptosis [31]. Impaired lipolysis and hypertrophy are hence powerful triggers of adipocyte apoptosis [32]. Lipotoxicity also triggers apoptosis of adipose tissue stem cells in aged WAT [33].

Adipocyte cell death is an inflammation-generating process, and is a prelude to a sequence of events leading to obesity-associated metabolic diseases [34]. In the setting of severe obesity, adipocyte apoptosis is prevalent and adipose tissue macrophages (ATMs) accumulate in the adipose tissue and form so-called crown-like structures around the dying adipocytes [35,36] (Figure 1c). As a result of the hypertrophic, lipid-overloaded adipocytes being larger than the ATMs, the ATMs fuse with each other to form a syncytial structure—a “giant”, multinucleated cell—that firmly encapsulates the dying adipocytes [37,38]. ATMs engulf cellular debris, lipid droplets, modified lipids, damage-associated molecules, apoptotic bodies, and fragments of adipocytes that are generated by secondary necrosis (Figure 1d) [39]. Unlike other tissues, the removal of these apoptotic cell contents triggers inflammation [6,17,37], and there is therefore a need for anti-inflammatory signals—including endocrine signals and Th2 cytokines—to equip ATMs with the ability to safely process adipocyte remnants [22,40]. Adipocyte death is hence an igniting event, which leads to pro-inflammatory ATM activation and adipose tissue inflammation, triggering further adipocyte apoptosis in a vicious cycle. A pro-inflammatory ATM phenotype is metabolically damaging, as it releases inflammatory mediators into the blood circulation, impedes insulin signaling, triggers meta-inflammation, initiates invasion of the adipose tissue with immune cells, and impairs the endocrine control of appetite and energy balance (Figure 1e) [12,41]. Inflammation also attracts monocytes and granulocytes to the adipose tissue, and these immune cells may also undergo apoptosis and their remnants also need to be cleared from the tissue to mitigate inflammation [34].

How macrophages respond to apoptotic adipocytes may depend on the immunological context in which adipocyte death occurs, the signals provided by the apoptotic cells [42], and additional immune signals such as complement and cytokines [43]. Under homeostatic conditions, when apoptotic adipocytes are scarce and Th2 cytokines are expressed in the WAT, the patrolling ATMs clear the dying adipocytes and adopt an M2-like activation state [44]. It is plausible that the first wave of apoptosis of adipocytes during the development of obesity is well controlled by the ATMs, and they are able to maintain an M2-like activation state [45]. However, the prevalence of damage-associated molecules and pro-inflammatory lipid species in the dying adipocytes can switch the function of ATMs towards the release of inflammatory cytokines and reactive oxygen species. Moreover, the capacity of ATMs to clear apoptotic cells may be exhausted in obesity [46], and the apoptotic or necrotic cell debris aggravates inflammation.

Inflammatory ATM traits are considered metabolically harmful [12]. However, inflammatory signaling is necessary for adipose tissue development, and inflammation coordinates energy distribution between tissues [47]. Paradoxically, when the response to pro-inflammatory cytokines is impaired, “inflammation resistance” develops, and the risk for obesity increases [47]. Type I interferon signaling, interleukin-6 (IL-6), and STAT3 signaling are required for healthy adipose tissue development and can be even protective against obesity-induced metabolic deterioration [23,48,49,50,51,52,53]. After birth, ATMs create a local inflammatory milieu, which sustains the heat production and lipid oxidation capacity of the adipocytes [23]. It is conceivable that preadipocyte or adipocyte apoptosis in the developing WAT induces ATM responses, which contribute to the maintenance of a local inflammatory signaling niche. Moreover, apoptotic cell-derived signals may promote WAT development, as suggested by the increased expression of adiponectin receptor 1 in response to conditioned medium of apoptotic cells [16]. ATMs are scarce in BAT [54,55], and the impact and mechanisms of apoptotic cell clearance in the BAT remain unexplored.

**Figure 2:**
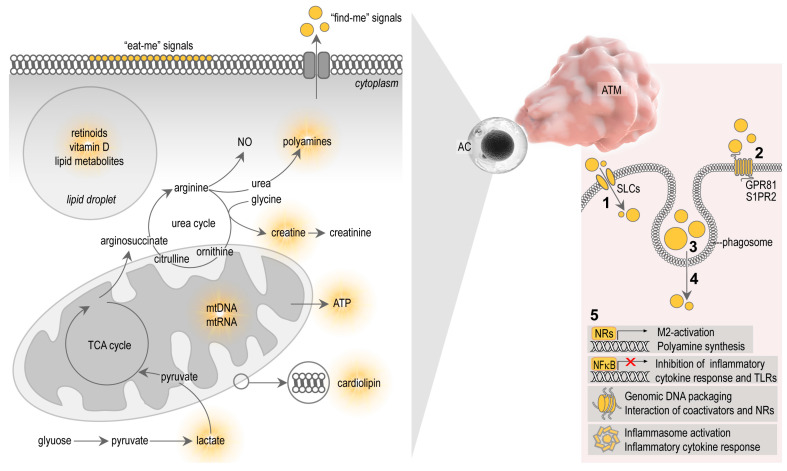
Apoptotic cell metabolites and their potential effects on macrophages. Apoptotic cells express cell surface markers which serve as “eat-me” signals, i.e., they initiate the binding and phagocytosis of the apoptotic cells by macrophages. Dying cells also actively release metabolites that attract macrophages, serving as “find-me” signals. Dying cells have various metabolites that potentially affect macrophage functions—for instance, lipid-soluble vitamins and lipid metabolites of lipid droplets and cell membranes, cytosolic metabolites such as polyamines, creatine, creatinine, ATP and lactate. Mitochondrial membranes and nucleic acids are also potent immune activators. (1) These metabolites may be taken-up by macrophages through solute carriers (SLCs). (2) Apoptotic cell metabolites can also activate G protein-linked membrane receptors in macrophages. (3) Apoptotic remnants are also phagocytosed by the macrophages and digested in phagosomes. (4) Efflux of metabolites from phagosomes may activate macrophage signaling pathways. (5) The macrophage response to apoptotic cells can be M2-activation and polyamine synthesis, and inhibition of NFκB and cytokine responses. Polyamines affect DNA packaging and the function of NRs. Some apoptotic contents can trigger inflammasome activation, inflammation and pyroptosis. TCA—tricarboxylic acid cycle, NO—nitric oxide, AC—apoptotic cell, ATM—adipose tissue macrophage, GPR 81—G protein-linked receptor 81, S1PR2—sphingosine 1-phosphate receptor 2, NR—nuclear receptor.

Signals that allow apoptotic cells to control macrophage behavior are complex, and involve diverse molecules and metabolites released by the apoptotic cells themselves. Apoptotic cells seem to actively elaborate cell metabolites, which serve as “find-me” signals, and display molecular patterns on the cell surfaces which serve as “eat-me” signals for the macrophages [42] (Figure 2). After ingestion of the apoptotic cells, cellular contents are lysed and processed in the phagosomes of the macrophages, ultimately producing signal molecules that control macrophage functions [56] (Figure 2). The study of the metabolites that shape immune response towards apoptotic cells is a new field of immunometabolism, recently termed as “efferotabolism” (efferocytosis-associated metabolism) [57]. Here, I review the possible metabolites of the apoptotic adipocytes that potentially control ATM behavior.

## 3. Metabolites of Apoptotic Cells and Their Effect on Macrophages

### 3.1. Nuclear Receptor Ligands

Microparticles released by apoptotic cells, and the apoptotic debris itself, contain membrane lipids and lipid mediators, which are metabolized in the macrophage phagosome to provide ligands for nuclear receptors (NRs) and trigger M2-activation (reviewed in [3,56], Figure 2). NRs are transcription factors that regulate gene transcription in a ligand-dependent manner [58]. Macrophages express several NRs, including peroxisome proliferator activator receptors (PPARs), liver X receptor (LXR), retinoid X receptor (RXR), retinoic acid receptor (RAR) and vitamin D receptor (VDR), and are all implicated in phagocytosis, a crucial mechanism for apoptotic cell clearance [59,60,61,62,63]. Apoptotic cells fail to inhibit pro-inflammatory cytokine responses in macrophages lacking PPARs or RXRs [62,63,64]. Furthermore, the absence of the PPARβ/δ ligand-binding domain is sufficient to impair M2-activation stimulated by apoptotic cells [62]. This suggests that PPAR/RXR signaling may be activated by lipid metabolites derived from cell membranes of the internalized apoptotic cells. However, the uptake of apoptotic cell membranes may also induce inflammasome activation in macrophages. Mitochondrial membranes, as well as entire mitochondria of the apoptotic cells, are released by apoptotic cells and trigger inflammasome activation due to the presence of damage-associated molecules in the apoptotic mitochondrial membrane, such as cardiolipin [65,66] (Figure 2).

Late phagosome functions are necessary to digest apoptotic cell membranes and make their lipid species accessible for NRs [62,63,67,68,69,70]. Engulfment of apoptotic cells by macrophages leads to the synthesis of retinoids, which, in part, are responsible for some transcriptional changes of the M2-activation program and for the sustained phagocytosis capacity of macrophages [68,69,71,72]. Retinoids accumulate in the adipocytes and can inhibit adipocyte differentiation, reduce fat accumulation, and promote the expression of uncoupling protein 1 (UCP1) and the development of BAT-like adipocytes in WAT depots in mouse [73,74,75,76,77]. Expression of UCP1 confers BAT-like properties to WAT adipocytes, including thermogenic potential—a process termed adipocyte browning or beige adipogenesis [78]. The resulting beige adipocytes have enhanced lipid catabolism and heat production ability, and they may help to burn-off stored lipids and mitigate obesity [10]. The subcutaneous adipose tissue of newborns is also rich in beige adipocytes and is thought to allow the efficient metabolism of lipid-rich breast milk, and to support the maintenance of body core temperature [23]. In contrast to mouse adipocytes, the beige adipocyte-inducing effect of retinoid signaling is lacking in human adipocytes [79]. Retinoids also induce apoptosis of adipose-derived stem cells [80], as well as of adipocytes of lean adipose tissue [81]. Adipocytes contain retinoids, are rich in free retinol, and express the enzymes necessary for vitamin A transport and metabolism (reviewed in [82]). The presence of retinoids in adipocytes likely means that ATMs engulfing adipocyte remnants are exposed to retinoids (Figure 2).

Similarly to retinoids, fat-soluble vitamin D is stored in adipocytes. The biologically active form of vitamin D, 1,25-dihydroxyvitamin D3, triggers apoptosis in mature adipocytes via induction of apoptotic Ca^2+^ signal [83], and high doses of dietary vitamin D intake leads to fat loss in mice [84]. Obese individuals can experience vitamin D deficiency [85,86], and VDR signaling has been reported to inhibit weight gain by activating muscle UCP3 [87]. VDR is also necessary for development of breast WAT [88]. Vitamin D biosynthesis is sunlight-dependent and seasonal light cycle changes are known to determine metabolic rate oscillations [89]. Indeed, a correlation exists between adult body mass index and season of birth, and some studies have reported an increased rate of obesity among winter-born individuals [90,91].

Vitamin D suppresses macrophage pro-inflammatory polarization in various settings [92,93,94]. For example, vitamin D supplementation attenuates inflammatory cytokine expression and promotes M2-macrophage traits in the epicardial adipose tissue of swine, whereas vitamin D deficiency exacerbates inflammation by increasing the number of pro-inflammatory macrophages [95]. Vitamin D also increases the expression of the antimicrobial peptide cathelicidin [96] and induces autophagy [93]. In the context of adipose tissue biology, cathelicidin is known to be expressed by the subcutaneous fat depot, and adipocyte autophagy is thought to mitigate obesity-associated diseases [97,98]. The intake of vitamin D from apoptotic adipocytes may affect ATM functions, potentially reducing pro-inflammatory cytokine expression, inducing autophagy, and aiding in the acquisition of antimicrobial traits (Figure 2).

### 3.2. Arginine and Polyamines

Apoptotic cells release metabolites that initiate cellular responses in neighboring cells and in macrophages that clear apoptotic debris. These signals are often called as “find-me” signals, since they promote the encounter between macrophages and apoptotic cells [42] and are released actively by dying cells through pannexin channels and possibly through other membrane channels [99].

An example of a “find-me” metabolite is sphingosine-1-phosphate, which binds to sphingosine-1-phosphate receptor 2 (S1PR2) to activate extracellular-signal-regulated kinase 5 (ERK5) and cAMP response element binding protein (CREB) signaling in macrophages [100]. These signaling cascades increase the expression of arginase 2 in macrophages, and hence increase arginine metabolism towards polyamines [101]. S1PR2 also reduces reactive oxygen species in macrophages [102]. Arginase 1 is a hallmark of M2-macrophages in the mouse, although pro-resolving macrophages may lack the increased expression of arginase 1 [103]. By contrast, arginase 2 is a ubiquitously expressed arginase enzyme [104]. Macrophages metabolize arginine into nitric oxide (NO) by NO synthases or may utilize it to produce polyamine precursors. This diversion of arginine metabolism is the so-called “arginine fork”, and conversion of arginine to NO is a trait of inflammatory macrophages, whereas metabolism of arginine in polyamine synthesis is a trait of M2-macrophages in the mouse [103]. Both arginase 1 and 2 can divert arginine metabolism from NO synthesis towards polyamine metabolism. Apoptotic cell-derived arginine is metabolized by macrophages to the polyamine putrescinein a process requiring arginase 1 and ornithine decarboxylase. Putrescine augments apoptotic cell uptake by inducing dynamic cytoskeletal changes [105]. S1PR2 has been reported to have anti-adipogenic effects [106]. For example, FTY720, a synthetic analog of sphingosine 1-phosphate, alleviates diet-induced obesity in mice, and reduces adipocyte hypertrophy and increases lipolysis [106]. Contrastingly, blocking S1PR2 signaling induces adipocyte proliferation but suppresses adipocyte differentiation [107].

Polyamines self-assemble with phosphate ions in the cell nucleus and generate so-called nuclear aggregates of polyamines, which interact with genomic DNA and control DNA conformation, protection and packaging [108]. Polyamines also affect the interactions of NRs with their coactivator complexes [109]. Given that NRs are important in both adipose tissue development and macrophage functions, especially in apoptotic cell removal (reviewed in [3]), it is plausible that polyamines shape macrophage functions in the process of efferocytosis at a transcriptional level (Figure 2).

### 3.3. Lactate

Lactate is a major circulating carbohydrate fuel in mammals [110] and adipocytes produce lactate from glucose [111]. Adipose tissue provides lactate for hepatic gluconeogenesis during fasting, as well as for hepatic glycogen synthesis after food ingestion [112]. The rate of glucose conversion to lactate increases with adipocyte size, and obese adipocytes may metabolize 50–70% of their glucose to lactate [111,113]. Lactate production is an anaerobic process, and adipose tissue is hypoxic under physiological conditions. Obesity increases adipose tissue hypoxia, favoring lactate production, and so obesity and diabetes are associated with markedly increased lactate production in adipocytes [112]. Moreover, lactate induces adipocyte browning in humans by controlling *UCP1* expression via intracellular redox modifications [114]. Lactate also reduces the circulating levels of free fatty acids as well as lipolysis in the adipose tissue [115].

Loss of mitochondrial membrane potential during apoptosis is associated with lactate production in some cell types [116]. In macrophages, efferocytosis also increases lactate release [16]. The underlying mechanism involves increased glucose uptake and enhanced glycolysis and lactate release through the solute carrier SLC16A [117]. The adipokine CTRP9, which enhances apoptotic cell uptake, also enhances the release of lactate from macrophages [16]. Lactate induces the expression of some M2-macrophage-associated genes, and triggers M2-like polarization [118,119]. Lactate also delays the proinflammatory response of human monocytes and mouse mast cells to lipopolysaccharide (LPS) [120,121] and reduces their secretion of TNFα [122]. Additionally, lactate suppresses NFκB signaling in macrophages and activates signaling through its cognate G protein-coupled receptor GPR81 [123,124] (reviewed in [125]). GPR81 is expressed in adipocytes and is responsible for the lactate-induced inhibition of lipolysis [115]. LPS signaling via Toll like receptor 4 (TLR4) was found to reduce *Gpr81* transcription in mouse WAT [126], making it plausible that impaired lactate signaling contributes to obesity. In summary, lactate is an underexplored metabolite of the adipose tissue with the potential to shape immune behavior of ATMs (Figure 2).

### 3.4. Creatine

Creatine is another metabolite, which is released by apoptotic cells and is enriched in the culture medium of dying cells. Macrophages are able to take up creatine through the solute career SLC6A8. Creatine suppresses pro-inflammatory macrophage activation, by impeding gene transcription induced by interferon gamma (IFNγ)-receptor signaling. Moreover, it promotes interleukin-4-induced M2-macrophage activation [127,128]. Creatinine is the breakdown product of creatine and, similarly to creatine, has effects on macrophages. Creatinine and creatine monohydrate suppress Toll like receptor (*Tlr2*, *Tlr3*, *Tlr4* and *Tlr7*) mRNA expression in the mouse macrophage-like leukemia cell line RAW 264.7 and in primary mouse splenocytes. Moreover, creatinine inhibits NFκB signaling and reduces basal-, and LPS-triggered TNFα production in macrophages [129] (Figure 2). By contrast, creatine ethyl ester increases the expression of *Tlr2*, *Tlr3*, *Tlr4* and *Tlr7* [130]. Creatine has a role in adipose tissue development and function: inhibiting creatine biosynthesis or deletion of the cell-surface creatine transporter in brown adipocytes reduces thermogenesis and causes obesity [131]. Creatine metabolism also increases beige adipogenesis and stimulates thermogenesis in WAT [132], and creatine supplementation during high-fat feeding increases energy expenditure in response to β3-adrenergic stimulation of beige adipogenesis [131]. As a food supplement, creatine appears to increase the loss of body fat during resistance training [133], although it does not improve glycemic control or reduce adipose tissue inflammation [134].

### 3.5. ATP and Nucleic Acids

Apoptotic cells show increased levels of cytosolic ATP, which is a prerequisite for apoptosis [135]. Apoptotic cells release ATP as a “find-me” signal, through pannexin 1 (ANX1) channels [136]. Pannexin-1 is one of three vertebrate pannexins that show homology to gap junction-forming invertebrate innexins [137] and has a role in the control of inflammation [138]. As a plasma membrane channel, ANX1 allows the release of “find-me” signals during apoptosis (reviewed in [99]). Stimulation of primary macrophages with ATP results in the production of high levels of reactive oxygen species and macrophage inflammatory protein-2, stimulating neutrophil migration [139]. ATP also induces inflammasome activation and pyroptosis in macrophages [140]. Activated macrophages also exocytose ATP, which, in turn, activates the ATP-sensor P2Y purinoceptor 11 (P2Y11) in macrophages in an autocrine mechanism, increasing inflammatory cytokine production [141]. Purinergic signaling has two separate roles in monocyte/macrophage activation—namely, to facilitate the initial detection of danger signals via TLRs and, subsequently, to regulate inflammasome activation [142].

Apoptotic cells may release mitochondrial remnants containing mitochondrial nucleic acids [66,143]. As mitochondrial RNA and DNA share some features of prokaryote RNA and DNA molecules, they can activate pathogen recognition receptors in macrophages. For example, apoptotic bodies contain a unique DNA species—5′ phosphorylated blunt-ended DNA—which is believed to shape the immune response to apoptotic cells [144]. Under physiological conditions, the released mitochondrial contents are safely recycled by mitophagy [145]; however, apoptosis results in the release of mitochondrial content into the cytosol [143]. Similarly, when mitophagy is impaired, there is a release of mitochondrial components into the cytosol, which triggers interferon response and inflammation [146]. In addition to membrane lipids, apoptotic bodies and apoptotic cells contain mitochondrial DNA, nuclear DNA fragments and various RNA species including double-stranded microRNAs, which are all potential inducers of inflammation and macrophage activation [66,147,148,149,150]. Indeed, the autoimmune disease systemic lupus erythematosus is characterized by self-immunity against nuclear fragments, which can be partly explained by the deficient clearance of apoptotic debris [151] or by aberrant apoptotic pathways [152]. It has recently been shown that apoptotic membrane vesicles are immunogenic in lupus erythematosus, possibly due to a deficiency in nucleic acid degradation during apoptosis [65,153]. The nucleic acids can activate the cytoplasmic DNA-sensing pathway (cGAS-STING signaling) and TLR7, TLR8 or TLR9 signaling [154], which induces the expression of interferon-stimulated genes [65,152,153]. Nucleic acids of the dying adipocytes are hence potential inducers of ATM activation and can trigger adipose tissue inflammation (Figure 2).

## 4. Summary and Perspective

**Figure 3:**
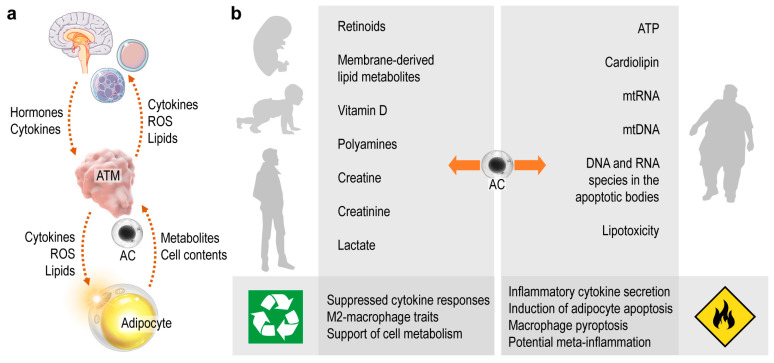
The two faces of apoptotic cell metabolites. (**a**) Chain of signaling events triggered by the apoptotic cell (AC). Metabolites and contents of the ACs are processed by ATMs. As a response, ATMs release cytokines, reactive oxygen species (ROS) and various lipid mediators, which signal to other immune cells, the central nervous system and the endocrine organs. In turn, these cells and organs release hormones, neurotransmitters and cytokines that shape ATM functions and, ultimately, determine the metabolic performance of the adipocytes. (**b**) Metabolites and contents of the dying adipocytes may be safely recycled by macrophages in an immunologically silent process, which results in gain of M2-macrophage traits and supports tissue turnover and metabolic health. Some contents of the apoptotic cells—especially of the dying adipocytes—deliver danger signals to the macrophages, and trigger inflammation and inflammatory cell death. Eventually the tissue integrity is impaired and immune-metabolic disease develops, such as meta-inflammation in the obese adipose tissue.

Beyond its traditional role as a lipid storage site, adipose tissue is increasingly recognized as being pivotal in metabolic and endocrine physiology [9]. ATMs are positioned at the interface between these functions, and interactions between ATMs and adipocytes potentially affect systemic metabolism and endocrine health (Figure 3a). Uptake of apoptotic cells is a core function of macrophages and serves as a communication channel between adipocytes and ATMs. The rich immunometabolism of apoptotic cell contents shapes ATM function, which in turn sustains healthy metabolism by balancing the protective and destructive immune functions of ATMs. Protective immunity ensures the neutralization of apoptotic cells and favors cell metabolism and energy expenditure (Figure 3a). When ATMs process materials of the dying adipocytes correctly, they signal to the endocrine organs and other immune cells, which ultimately supports adipose tissue development and metabolism (Figure 3a). By contrast, destructive immune traits appear in response to danger signals from dying adipocytes (Figure 3b). The resulting ATM phenotype damages metabolism through uncontrolled inflammation or self-immunity [155], leading to a dysfunctional interplay between ATMs and adipocytes and driving the development of metabolic diseases [155,156]. (Figure 3b). As adipocyte death is prevalent in obese adipose tissue, the majority of the literature has focused on the role of ATMs in the setting of obesity. However, the immune functioning of ATMs begins at birth, and is crucial to sustain energy expenditure in infancy [23,52,155,157]. Adipocyte apoptosis may occur in the adipose tissue of the neonate, but we know very little about the impact of ATM-adipocyte interactions in early postnatal development, even though adipose tissue quality in infancy is contingent on ATM function and determines metabolic health in adulthood [23,158]. It is thus important to comprehend how physiological adipocyte death (i.e., during adipose tissue development) shapes ATM functions in postnatal life.

Several signals that potentially define whether ATMs safely recycle adipocyte contents or trigger inflammation are known and have been reviewed here (Figure 3b). In the coming decades the treatment of obesity and associated chronic diseases will likely remain a major challenge to health systems worldwide; thus, it is timely and important to understand the impact of innate immune signaling—including apoptotic adipocyte-derived signals—in the adipose tissue.

## Data Availability

Not applicable.

## References

[1] Poon I., Lucas C., Rossi A.G., Ravichandran K. (2014). Apoptotic cell clearance: Basic biology and therapeutic potential. Nat. Rev. Immunol..

[2] Kourtzelis I., Hajishengallis G., Chavakis T. (2020). Phagocytosis of Apoptotic Cells in Resolution of Inflammation. Front. Immunol..

[3] Röszer T. (2016). Transcriptional control of apoptotic cell clearance by macrophage nuclear receptors. Apoptosis.

[4] Röszer T. (2020). Signal Mechanisms of M2 Macrophage Activation. The M2 Macrophage.

[5] Zizzo G., Hilliard B.A., Monestier M., Cohen P.L. (2012). Efficient Clearance of Early Apoptotic Cells by Human Macrophages Requires M2c Polarization and MerTK Induction. J. Immunol..

[6] Lindhorst A., Raulien N., Wieghofer P., Eilers J., Rossi F.M.V., Bechmann I., Gericke M. (2021). Adipocyte death triggers a pro-inflammatory response and induces metabolic activation of resident macrophages. Cell Death Dis..

[7] Hahn P., Novak M. (1975). Development of brown and white adipose tissue. J. Lipid Res..

[8] Hull D. (1966). The structure and function of brown adipose tissue. Br. Med. Bull..

[9] Rosen E.D., Spiegelman B.M. (2014). What We Talk About When We Talk About Fat. Cell.

[10] Harms M., Seale P. (2013). Brown and beige fat: Development, function and therapeutic potential. Nat. Med..

[11] Bartelt A., Heeren J. (2013). Adipose tissue browning and metabolic health. Nat. Rev. Endocrinol..

[12] Boutens L., Stienstra R. (2016). Adipose tissue macrophages: Going off track during obesity. Diabetologia.

[13] Sorisky A., Magun R., Gagnon A. (2000). Adipose cell apoptosis: Death in the energy depot. Int. J. Obes..

[14] Hirsch J., Faust I.M., Johnson P.R. (1979). What’s New in Obesity: Current Understanding of Adipose Tissue. Morphology.

[15] Nisoli E., Cardile A., Bulbarelli A., Tedesco L., Bracale R., Cozzi V., Morroni M., Cinti S., Valerio A., Carruba M.O. (2006). White adipocytes are less prone to apoptotic stimuli than brown adipocytes in rodent. Cell Death Differ..

[16] Song C.-X., Chen J.-Y., Li N., Guo Y. (2021). CTRP9 Enhances Efferocytosis in Macrophages via MAPK/Drp1-Mediated Mitochondrial Fission and AdipoR1-Induced Immunometabolism. J. Inflamm. Res..

[17] Kuroda M., Sakaue H. (2017). Adipocyte Death and Chronic Inflammation in Obesity. J. Med. Investig..

[18] McLaughlin T., Craig C., Liu L.-F., Perelman D., Allister C., Spielman D., Cushman S.W. (2016). Adipose Cell Size and Regional Fat Deposition as Predictors of Metabolic Response to Overfeeding in Insulin-Resistant and Insulin-Sensitive Humans. Diabetes.

[19] Björntorp P., Sjöström L. (1972). The Composition and Metabolism in Vitro of Adipose Tissue Fat Cells of Different Sizes. Eur. J. Clin. Investig..

[20] Boulton T.J.C., Dunlop M., Court J.M. (2008). Adipocyte growth in the first 2 years of life. J. Paediatr. Child. Health.

[21] Dauncey M.J., Gairdner D. (1975). Size of adipose cells in infancy. Arch. Dis. Child..

[22] Waqas S.F.H., Hoang A.C., Lin Y.-T., Ampem G., Azegrouz H., Balogh L., Thuróczy J., Chen J.-C., Gerling I.C., Nam S. (2017). Neuropeptide FF increases M2 activation and self-renewal of adipose tissue macrophages. J. Clin. Investig..

[23] Yu H., Dilbaz S., Coßmann J., Hoang A.C., Diedrich V., Herwig A., Harauma A., Hoshi Y., Moriguchi T., Landgraf K. (2019). Breast milk alkylglycerols sustain beige adipocytes through adipose tissue macrophages. J. Clin. Investig..

[24] Vogel P., Read R., Hansen G., Wingert J., Dacosta C.M., Buhring L.M., Shadoan M. (2011). Pathology of congenital generalized lipodystrophy in Agpat2-/- mice. Vet. Pathol..

[25] Birk R.Z., Rubinstein M. (2006). IFN-α induces apoptosis of adipose tissue cells. Biochem. Biophys. Res. Commun..

[26] Domingo P., Matias-Guiu X., Pujol R.M., Francia E., Lagarda E., Sambeat M.A., Vázquez G. (1999). Subcutaneous adipocyte apoptosis in HIV-1 protease inhibitor-associated lipodystrophy. AIDS.

[27] Alcalá M., Calderon-Dominguez M., Bustos E., Ramos P., Casals N., Serra D., Viana M., Herrero L. (2017). Increased inflammation, oxidative stress and mitochondrial respiration in brown adipose tissue from obese mice. Sci. Rep..

[28] Zhang H.H., Kumar S., Barnett A.H., Eggo M.C. (2001). Dexamethasone inhibits tumor necrosis factor-alpha-induced apoptosis and interleukin-1 beta release in human subcutaneous adipocytes and preadipocytes. J. Clin. Endocrinol. Metab..

[29] Tchkonia T., Tchoukalova Y.D., Giorgadze N., Pirtskhalava T., Karagiannides I., Forse R.A., Koo A., Stevenson M., Chinnappan D., Cartwright A. (2005). Abundance of two human preadipocyte subtypes with distinct capacities for replication, adipogenesis, and apoptosis varies among fat depots. Am. J. Physiol. Metab..

[30] Prieur X., Rőszer T., Ricote M. (2010). Lipotoxicity in macrophages: Evidence from diseases associated with the metabolic syndrome. Biochim. Biophys. Acta Mol. Cell Biol. Lipids.

[31] Qian S., Pan J., Su Y., Tang Y., Wang Y., Zou Y., Zhao Y., Ma H., Zhang Y., Liu Y. (2020). BMPR2 promotes fatty acid oxidation and protects white adipocytes from cell death in mice. Commun. Biol..

[32] Osuga J.-I., Ishibashi S., Oka T., Yagyu H., Tozawa R., Fujimoto A., Shionoiri F., Yahagi N., Kraemer F., Tsutsumi O. (2000). Targeted disruption of hormone-sensitive lipase results in male sterility and adipocyte hypertrophy, but not in obesity. Proc. Natl. Acad. Sci. USA.

[33] Guo W., Pirtskhalava T., Tchkonia T., Xie W., Thomou T., Han J., Wang T., Wong S., Cartwright A., Hegardt F.G. (2007). Aging results in paradoxical susceptibility of fat cell progenitors to lipotoxicity. Am. J. Physiol. Metab..

[34] West M. (2009). Dead adipocytes and metabolic dysfunction: Recent progress. Curr. Opin. Endocrinol. Diabetes Obes..

[35] Xu H., Barnes G.T., Yang Q., Tan G., Yang D., Chou C.J., Sole J., Nichols A., Ross J.S., Chen H. (2003). Chronic inflammation in fat plays a crucial role in the development of obesity-related insulin resistance. J. Clin. Investig..

[36] Cinti S., Mitchell G., Barbatelli G., Murano I., Ceresi E., Faloia E., Wang S., Fortier M., Greenberg A.S., Obin M.S. (2005). Adipocyte death defines macrophage localization and function in adipose tissue of obese mice and humans. J. Lipid Res..

[37] Lumeng C.N., Bodzin J.L., Saltiel A.R. (2007). Obesity induces a phenotypic switch in adipose tissue macrophage polarization. J. Clin. Investig..

[38] Braune J., Lindhorst A., Fröba J., Hobusch C., Kovacs P., Blüher M., Eilers J., Bechmann I., Gericke M. (2020). Multinucleated Giant Cells in Adipose Tissue Are Specialized in Adipocyte Degradation. Diabetes.

[39] Strissel K.J., Stancheva Z., Miyoshi H., Perfield J.W., DeFuria J., Jick Z., Greenberg A.S., Obin M.S. (2007). Adipocyte death, adipose tissue remodeling, and obesity complications. Diabetes.

[40] Dai L., Bhargava P., Stanya K.J., Alexander R.K., Liou Y.-H., Jacobi D., Knudsen N.H., Hyde A., Gangl M.R., Liu S. (2017). Macrophage alternative activation confers protection against lipotoxicity-induced cell death. Mol. Metab..

[41] Nagareddy P., Kraakman M., Masters S., Stirzaker R.A., Gorman D.J., Grant R., Dragoljevic D., Hong E.S., Abdel-Latif A., Smyth S.S. (2014). Adipose Tissue Macrophages Promote Myelopoiesis and Monocytosis in Obesity. Cell Metab..

[42] Ravichandran K.S. (2010). Find-me and eat-me signals in apoptotic cell clearance: Progress and conundrums. J. Exp. Med..

[43] Benoit M.E., Clarke E., Morgado P., Fraser D.A., Tenner A.J. (2012). Complement Protein C1q Directs Macrophage Polarization and Limits Inflammasome Activity during the Uptake of Apoptotic Cells. J. Immunol..

[44] Fischer-Posovszky P., Wang Q., Asterholm I.W., Rutkowski J., Scherer P.E. (2011). Targeted Deletion of Adipocytes by Apoptosis Leads to Adipose Tissue Recruitment of Alternatively Activated M2 Macrophages. Endocrinology.

[45] Shaul M.E., Bennett G., Strissel K.J., Greenberg A.S., Obin M.S. (2010). Dynamic, M2-Like Remodeling Phenotypes of CD11c+ Adipose Tissue Macrophages During High-Fat Diet-Induced Obesity in Mice. Diabetes.

[46] Luo B., Wang Z., Zhang Z.-Y., Shen Z., Zhang Z. (2018). The deficiency of macrophage erythropoietin signaling contributes to delayed acute inflammation resolution in diet-induced obese mice. Biochim. Biophys. Acta (BBA)—Mol. Basis Dis..

[47] Wang H., Ye J. (2014). Regulation of energy balance by inflammation: Common theme in physiology and pathology. Rev. Endocr. Metab. Disord..

[48] Wieser V., Adolph T.E., Grander C., Grabherr F., Enrich B., Moser P., Moschen A.R., Kaser S., Tilg H. (2016). Adipose type I interferon signalling protects against metabolic dysfunction. Gut.

[49] Derecka M., Gornicka A., Koralov S.B., Szczepanek K., Morgan M., Raje V., Sisler J., Zhang Q., Otero D., Cichy J. (2012). Tyk2 and Stat3 Regulate Brown Adipose Tissue Differentiation and Obesity. Cell Metab..

[50] Kristóf E., Klusóczki A., Veress R., Shaw A., Combi Z.S., Varga K., Győry F., Balajthy Z., Bai P., Bacso Z. (2019). Interleukin-6 released from differentiating human beige adipocytes improves browning. Exp. Cell Res..

[51] Alsaggar M., Mills M., Liu D. (2016). Interferon beta overexpression attenuates adipose tissue inflammation and high-fat diet-induced obesity and maintains glucose homeostasis. Gene Ther..

[52] Babaei R., Schuster M., Meln I., Lerch S., Ghandour R.A., Pisani D.F., Bayindir-Buchhalter I., Marx J., Wu S., Schoiswohl G. (2018). Jak-TGFβ cross-talk links transient adipose tissue inflammation to beige adipogenesis. Sci. Signal..

[53] Asterholm I.W., Tao C., Morley T.S., Wang Q., Delgado-Lopez F., Wang Z., Scherer P.E. (2014). Adipocyte Inflammation Is Essential for Healthy Adipose Tissue Expansion and Remodeling. Cell Metab..

[54] Waqas S.F.H., Noble A., Hoang A.C., Ampem G., Popp M., Strauß S., Guille M., Röszer T. (2017). Adipose tissue macrophages develop from bone marrow–independent progenitors in Xenopus laevis and mouse. J. Leukoc. Biol..

[55] Peterson K.R., Flaherty D.K., Hasty A.H. (2017). Obesity Alters B Cell and Macrophage Populations in Brown Adipose Tissue. Obesity.

[56] Han C.Z., Ravichandran K.S. (2011). Metabolic Connections during Apoptotic Cell Engulfment. Cell.

[57] Yurdagul A. (2021). Metabolic consequences of efferocytosis and its impact on atherosclerosis. Immunometabolism.

[58] Röszer T., Menéndez-Gutiérrez M.P., Cedenilla M., Ricote M. (2013). Retinoid X receptors in macrophage biology. Trends Endocrinol. Metab..

[59] Vasina E.M., Cauwenberghs S., Feijge M.A.H., Heemskerk J.W.M., Weber C., Koenen R.R. (2011). Microparticles from apoptotic platelets promote resident macrophage differentiation. Cell Death Dis..

[60] Rigamonti E., Chinetti-Gbaguidi G., Staels B. (2008). Regulation of macrophage functions by PPAR-alpha, PPAR-gamma, and LXRs in mice and men. Arterioscler. Thromb. Vasc. Biol..

[61] Gonzalez N.A., Bensinger S.J., Hong C., Beceiro S., Bradley M.N., Zelcer N., Deniz J., Ramirez C., Díaz M., Castrillo A. (2009). Apoptotic cells promote their own clearance and immune tolerance through activation of the nuclear receptor LXR. Immunity.

[62] Mukundan L., Odegaard J.I., Morel C.R., Heredia J.E., Mwangi J.W., Ricardo-Gonzalez R.R., Goh Y.P.S., Eagle A.R., Dunn S.E., Chawla A. (2009). PPAR-delta senses and orchestrates clearance of apoptotic cells to promote tolerance. Nat. Med..

[63] Röszer T., Menéndez-Gutiérrez M.P., Lefterova M.I., Alameda D., Núñez V., Lazar M.A., Fischer T., Ricote M. (2011). Autoimmune kidney disease and impaired engulfment of apoptotic cells in mice with macrophage peroxisome proliferator-activated receptor gamma or retinoid X receptor alpha deficiency. J. Immunol..

[64] Yoon Y.S., Kim S.-Y., Kim M.-J., Lim J.-H., Cho M.-S., Kang J.L. (2015). PPARgamma activation following apoptotic cell instillation promotes resolution of lung inflammation and fibrosis via regulation of efferocytosis and proresolving cytokines. Mucosal. Immunol..

[65] Kato Y., Park J., Takamatsu H., Konaka H., Aoki W., Aburaya S., Ueda M., Nishide M., Koyama S., Kumanogoh A. (2018). Apoptosis-derived membrane vesicles drive the cGAS-STING pathway and enhance type I IFN production in systemic lupus erythematosus. Ann. Rheum. Dis..

[66] Zhu M., Barbas A.S., Lin L., Scheuermann U., Bishawi M., Brennan T.V. (2018). Mitochondria Released by Apoptotic Cell Death Initiate Innate Immune Responses. ImmunoHorizons.

[67] Penberthy K., Ravichandran K.S. (2015). Apoptotic cell recognition receptors and scavenger receptors. Immunol. Rev..

[68] Garabuczi E., Kiss B., Felszeghy S.B., Tsay G.J., Fésüs L., Szondy Z. (2011). Retinoids produced by macrophages engulfing apoptotic cells contribute to the appearance of transglutaminase 2 in apoptotic thymocytes. Amino Acids.

[69] Sarang Z., Garabuczi É., Joós G., Kiss B., Tóth K., Rühl R., Szondy Z. (2013). Macrophages engulfing apoptotic thymocytes produce retinoids to promote selection, differentiation, removal and replacement of double positive thymocytes. Immunobiology.

[70] Rébé C., Raveneau M., Chevriaux A., Lakomy D., Sberna A.-L., Costa A., Bessède G., Athias A., Steinmetz E., Lobaccaro J.M.A. (2009). Induction of Transglutaminase 2 by a Liver X Receptor/Retinoic Acid Receptor α Pathway Increases the Clearance of Apoptotic Cells by Human Macrophages. Circ. Res..

[71] Tóth B., Garabuczi É., Sarang Z., Vereb G., Vámosi G., Aeschlimann D., Blaskó B., Bécsi B., Erdõdi F., Lacy-Hulbert A. (2009). Transglutaminase 2 Is Needed for the Formation of an Efficient Phagocyte Portal in Macrophages Engulfing Apoptotic Cells. J. Immunol..

[72] Sarang Z., Joós G., Garabuczi É., Rühl R., Gregory C.D., Szondy Z. (2014). Macrophages Engulfing Apoptotic Cells Produce Nonclassical Retinoids To Enhance Their Phagocytic Capacity. J. Immunol..

[73] Flajollet S., Staels B., Lefebvre P. (2013). Retinoids and nuclear retinoid receptors in white and brown adipose tissues: Physiopathologic aspects. Horm. Mol. Biol. Clin. Investig..

[74] Teruel T., Hernandez R., Benito M., Lorenzo M. (2003). Rosiglitazone and Retinoic Acid Induce Uncoupling Protein-1 (UCP-1) in a p38 Mitogen-activated Protein Kinase-dependent Manner in Fetal Primary Brown Adipocytes. J. Biol. Chem..

[75] Mercader J., Ribot J., Murano I., Felipe F., Cinti S., Bonet M.L., Palou A. (2006). Remodeling of White Adipose Tissue after Retinoic Acid Administration in Mice. Endocrinology.

[76] Jeyakumar S.M., Vajreswari A., Giridharan N.V. (2006). Chronic Dietary Vitamin A Supplementation Regulates Obesity in an Obese Mutant WNIN/Ob Rat Model. Obesity.

[77] Tan L., Zhang Y., Crowe-White K.M., Senkus K.E., Erwin M.E., Wang H. (2020). Vitamin A supplementation during suckling and postweaning periods attenuates the adverse metabolic effects of maternal high-fat diet consumption in Sprague-Dawley Rats. Curr. Dev. Nutr..

[78] Sidossis L., Kajimura S. (2015). Brown and beige fat in humans: Thermogenic adipocytes that control energy and glucose homeostasis. J. Clin. Investig..

[79] Murholm M., Isidor M.S., Basse A.L., Winther S., Sørensen C., Skovgaard-Petersen J., Nielsen M.M., Hansen A.S., Quistorff B., Hansen J.B. (2013). Retinoic acid has different effects on UCP1 expression in mouse and human adipocytes. BMC Cell Biol..

[80] Schweich L.D.C., De Oliveira E.J.T., Pesarini J.R., Hermeto L.C., Camassola M., Nardi N.B., Brochado T.M.M., Antoniolli-Silva A.C.M.B., Oliveira R.J. (2017). All-trans retinoic acid induces mitochondria-mediated apoptosis of human adipose-derived stem cells and affects the balance of the adipogenic differentiation. Biomed. Pharmacother..

[81] Jeyakumar S.M., Vajreswari A., Sesikeran B., Giridharan N.V. (2005). Vitamin A supplementation induces adipose tissue loss through apoptosis in lean but not in obese rats of the WNIN/Ob strain. J. Mol. Endocrinol..

[82] Landrier J.-F., Marcotorchino J., Tourniaire F. (2012). Lipophilic Micronutrients and Adipose Tissue Biology. Nutrients.

[83] Sergeev I.N. (2014). Vitamin D-mediated apoptosis in cancer and obesity. Horm. Mol. Biol. Clin. Investig..

[84] Sergeev I.N., Song Q. (2014). High vitamin D and calcium intakes reduce diet-induced obesity in mice by increasing adipose tissue apoptosis. Mol. Nutr. Food Res..

[85] De Oliveira L.F., de Azevedo L.G., da Mota Santana J., de Sales L.P.C., Pereira-Santos M. (2020). Obesity and overweight decreases the effect of vitamin D supplementation in adults: Systematic review and meta-analysis of randomized controlled trials. Rev. Endocr. Metab. Dis..

[86] Pramono A., Jocken J.W., Blaak E.E. (2019). Vitamin D deficiency in the aetiology of obesity-related insulin resistance. Diabetes/Metabolism Res. Rev..

[87] Fan Y., Futawaka K., Koyama R., Fukuda Y., Hayashi M., Imamoto M., Miyawaki T., Kasahara M., Tagami T., Moriyama K. (2016). Vitamin D3/VDR resists diet-induced obesity by modulating UCP3 expression in muscles. J. Biomed. Sci..

[88] Narvaez C.J., Matthews D., Broun E., Chan M., Welsh J. (2009). Lean Phenotype and Resistance to Diet-Induced Obesity in Vitamin D Receptor Knockout Mice Correlates with Induction of Uncoupling Protein-1 in White Adipose Tissue. Endocrinology.

[89] Diedrich V., Haugg E., Dreier C., Herwig A. (2020). What can seasonal models teach us about energy balance?. J. Endocrinol..

[90] Wattie N., Ardern C.I., Baker J. (2008). Season of birth and prevalence of overweight and obesity in Canada. Early Hum. Dev..

[91] Phillips D., Young J. (2000). Birth weight, climate at birth and the risk of obesity in adult life. Int. J. Obes..

[92] Wasnik S., Rundle C.H., Baylink D.J., Yazdi M.S., Carreon E.E., Xu Y., Qin X., Lau K.-H.W., Tang X. (2018). 1,25-Dihydroxyvitamin D suppresses M1 macrophages and promotes M2 differentiation at bone injury sites. JCI Insight.

[93] Das L.M., Binko A.M., Traylor Z.P., Peng H., Lu K.Q. (2019). Vitamin D improves sunburns by increasing autophagy in M2 macrophages. Autophagy.

[94] Zhang Y., Leung D.Y.M., Richers B.N., Liu Y., Remigio L.K., Riches D.W., Goleva E. (2012). Vitamin D Inhibits Monocyte/Macrophage Proinflammatory Cytokine Production by Targeting MAPK Phosphatase-1. J. Immunol..

[95] Gunasekar P., Swier V.J., Fleegel J.P., Boosani C., Radwan M.M., Agrawal D.K. (2018). Vitamin D and macrophage polarization in epicardial adipose tissue of atherosclerotic swine. PLoS ONE.

[96] Zhang L.-J., Guerrero-Juarez C.F., Hata T., Bapat S.P., Ramos R., Plikus M.V., Gallo R.L. (2015). Dermal adipocytes protect against invasive *Staphylococcus aureus* skin infection. Science.

[97] Fernández Á.F., Bárcena C., Martínez-García G.G., Tamargo-Gómez I., Suárez M.F., Pietrocola F., Mariño G. (2017). Autophagy couteracts weight gain, lipotoxicity and pancreatic β-cell death upon hypercaloric pro-diabetic regimens. Cell Death Dis..

[98] Rosa-Caldwell M.E., Brown J.L., Lee D.E., Blackwell T.A., Turner K.W., Brown L.A., Perry R.A., Haynie W.S., Washington T.A., Greene N.P. (2017). Autophagy activation, not peroxisome proliferator-activated receptor γ coactivator 1α, may mediate exercise-induced improvements in glucose handling during diet-induced obesity. Exp. Physiol..

[99] Chekeni F.B., Elliott M., Sandilos J.K., Walk S.F., Kinchen J., Lazarowski E.R., Armstrong A.J., Penuela S., Laird D.W., Salvesen G.S. (2010). Pannexin 1 channels mediate ‘find-me’ signal release and membrane permeability during apoptosis. Nature.

[100] Medina C.B., Mehrotra P., Arandjelovic S., Perry J.S.A., Guo Y., Morioka S., Barron B., Walk S.F., Ghesquière B., Krupnick A.S. (2020). Metabolites released from apoptotic cells act as tissue messengers. Nature.

[101] Barra V., Kuhn A.-M., Von Knethen A., Weigert A., Brüne B. (2010). Apoptotic cell-derived factors induce arginase II expression in murine macrophages by activating ERK5/CREB. Cell. Mol. Life Sci..

[102] Herr D.R., Reolo M.J.Y., Peh Y.X., Wang W., Lee C.-W., Rivera R., Paterson I.C., Chun J. (2016). Sphingosine 1-phosphate receptor 2 (S1P2) attenuates reactive oxygen species formation and inhibits cell death: Implications for otoprotective therapy. Sci. Rep..

[103] Röszer T., Röszer T. (2020). What Is an M2 Macrophage? Historical Overview of the Macrophage Polarization Model. The Th1/Th2 and M1/M2 Paradigm, the Arginine Fork. The M2 Macrophage.

[104] Rőszer T. (2015). Understanding the Mysterious M2 Macrophage through Activation Markers and Effector Mechanisms. Mediat. Inflammation.

[105] Yurdagul A., Subramanian M., Wang X., Crown S.B., Ilkayeva O.R., Darville L., Kolluru G.K., Rymond C.C., Gerlach B.D., Zheng Z. (2020). Macrophage Metabolism of Apoptotic Cell-Derived Arginine Promotes Continual Efferocytosis and Resolution of Injury. Cell Metab..

[106] Moon M.H., Jeong J.K., Park S.Y. (2015). Activation of S1P2 receptor, a possible mechanism of inhibition of adipogenic differentiation by sphingosine 1-phosphate. Mol. Med. Rep..

[107] Kitada Y., Kajita K., Taguchi K., Mori I., Yamauchi M., Ikeda T., Kawashima M., Asano M., Kajita T., Ishizuka T. (2016). Blockade of Sphingosine 1-Phosphate Receptor 2 Signaling Attenuates High-Fat Diet-Induced Adipocyte Hypertrophy and Systemic Glucose Intolerance in Mice. Endocrinology.

[108] Iacomino G., Picariello G., D’Agostino L. (2012). DNA and nuclear aggregates of polyamines. Biochim. Biophys. Acta (BBA)—Mol. Cell Res..

[109] Maeda Y., Rachez C., Hawel L., Byus C.V., Freedman L.P., Sladek F.M. (2002). Polyamines Modulate the Interaction between Nuclear Receptors and Vitamin D Receptor-Interacting Protein 205. Mol. Endocrinol..

[110] Rabinowitz J.D., Enerbäck S. (2020). Lactate: The ugly duckling of energy metabolism. Nat. Metab..

[111] Krycer J.R., Quek L.-E., Francis D., Fazakerley D.J., Elkington S.D., Diaz-Vegas A., Cooke K.C., Weiss F.C., Duan X., Kurdyukov S. (2020). Lactate production is a prioritized feature of adipocyte metabolism. J. Biol. Chem..

[112] DiGirolamo M., Newby F.D., Lovejoy J. (1992). Lactate production in adipose tissue; a regulated function with extra-adipose implications. FASEB J..

[113] Muñoz S., Franckhauser S., Elias I., Ferre T., Hidalgo A., Monteys A.M., Molas M., Cerdan S., Pujol A., Ruberte J. (2010). Chronically increased glucose uptake by adipose tissue leads to lactate production and improved insulin sensitivity rather than obesity in the mouse. Diabetologia.

[114] Carrière A., Jeanson Y., Berger-Müller S., André M., Chenouard V., Arnaud E., Barreau C., Walther R., Galinier A., Wdziekonski B. (2014). Browning of White Adipose Cells by Intermediate Metabolites: An Adaptive Mechanism to Alleviate Redox Pressure. Diabetes.

[115] Cai T.-Q., Ren N., Jin L., Cheng K., Kash S., Chen R., Wright S.D., Taggart A.K., Waters M.G. (2008). Role of GPR81 in lactate-mediated reduction of adipose lipolysis. Biochem. Biophys. Res. Commun..

[116] Tiefenthaler M., Amberger A., Bacher N., Hartmann B.L., Margreiter R., Kofler R., Konwalinka G. (2001). Increased lactate production follows loss of mitochondrial membrane potential during apoptosis of human leukaemia cells. Br. J. Haematol..

[117] Morioka S., Perry J.S.A., Raymond M.H., Medina C.B., Zhu Y., Zhao L., Serbulea V., Onengut-Gumuscu S., Leitinger N., Kucenas S. (2018). Efferocytosis induces a novel SLC program to promote glucose uptake and lactate release. Nature.

[118] Colegio O., Chu N.-Q., Szabo A.L., Chu T., Rhebergen A.M., Jairam V., Cyrus N., Brokowski C.E., Eisenbarth S., Phillips G.M. (2014). Functional polarization of tumour-associated macrophages by tumour-derived lactic acid. Nature.

[119] Selleri S., Bifsha P., Civini S., Pacelli C., Dieng M.M., Lemieux W., Jin P., Bazin R., Patey N., Marincola F.M. (2016). Human mesenchymal stromal cell-secreted lactate induces M2-macrophage differentiation by metabolic reprogramming. Oncotarget.

[120] Peter K., Rehli M., Singer K., Renner-Sattler K., Kreutz M. (2015). Lactic acid delays the inflammatory response of human monocytes. Biochem. Biophys. Res. Commun..

[121] Caslin H., Abebayehu D., Qayum A.A., Haque T.T., Taruselli M., Paez P.A., Pondicherry N., Barnstein B.O., Hoeferlin L.A., Chalfant C.E. (2019). Lactic Acid Inhibits Lipopolysaccharide-Induced Mast Cell Function by Limiting Glycolysis and ATP Availability. J. Immunol..

[122] Dietl K., Renner K., Dettmer K., Timischl B., Eberhart K., Dorn C., Hellerbrand C., Kastenberger M., Kunz-Schughart L., Oefner P.J. (2009). Lactic Acid and Acidification Inhibit TNF Secretion and Glycolysis of Human Monocytes. J. Immunol..

[123] Yang K., Xu J., Fan M., Tu F., Wang X., Ha T., Williams D.L., Li C. (2020). Lactate Suppresses Macrophage Pro-Inflammatory Response to LPS Stimulation by Inhibition of YAP and NF-κB Activation via GPR81-Mediated Signaling. Front. Immunol..

[124] Hoque R., Farooq A., Ghani A., Gorelick F., Mehal W.Z. (2014). Lactate Reduces Liver and Pancreatic Injury in Toll-Like Receptor—And Inflammasome-Mediated Inflammation via GPR81-Mediated Suppression of Innate Immunity. Gastroenterology.

[125] Zhou H.-C., Yan X.-Y., Yu W.-W., Liang X.-Q., Du X.-Y., Liu Z.-C., Long J.-P., Zhao G.-H., Liu H.-B. (2021). Lactic acid in macrophage polarization: The significant role in inflammation and cancer. Int. Rev. Immunol..

[126] Feingold K.R., Moser A., Shigenaga J.K., Grunfeld C. (2011). Inflammation inhibits GPR81 expression in adipose tissue. Inflamm. Res..

[127] Ji L., Zhao X., Zhang B., Kang L., Song W., Zhao B., Xie W., Chen L., Hu X. (2019). Slc6a8-Mediated Creatine Uptake and Accumulation Reprogram Macrophage Polarization via Regulating Cytokine Responses. Immunity.

[128] Ji L., Zhao X., Zhang B., Kang L., Song W., Zhao B., Xie W., Hu X. (2019). Creatine shapes macrophage polarization by reprogramming L-arginine metabolism. J. Immunol..

[129] Riesberg L.A., McDonald T.L., Wang Y., Chen X.-M., Holzmer S.W., Tracy S.M., Drescher K.M. (2018). Creatinine downregulates TNF-α in macrophage and T cell lines. Cytokine.

[130] Leland K.M., McDonald T.L., Drescher K.M. (2011). Effect of creatine, creatinine, and creatine ethyl ester on TLR expression in macrophages. Int. Immunopharmacol..

[131] Kazak L., Rahbani J., Samborska B., Lu G.Z., Jedrychowski M.P., Lajoie M., Zhang S., Ramsay L., Dou F., Tenen D. (2019). Ablation of adipocyte creatine transport impairs thermogenesis and causes diet-induced obesity. Nat. Metab..

[132] Kazak L., Chouchani E.T., Jedrychowski M.P., Erickson B., Shinoda K., Cohen P., Vetrivelan R., Lu G.Z., Laznik-Bogoslavski D., Hasenfuss S.C. (2015). A Creatine-Driven Substrate Cycle Enhances Energy Expenditure and Thermogenesis in Beige Fat. Cell.

[133] Forbes S.C., Candow D.G., Krentz J.R., Roberts M.D., Young K.C. (2019). Changes in Fat Mass Following Creatine Supplementation and Resistance Training in Adults ≥50 Years of Age: A Meta-Analysis. J. Funct. Morphol. Kinesiol..

[134] Oliveira C.L., Antunes B.D.M.M., Gomes A.C., Lira F.S., Pimentel G.D., Boulé N.G., Mota J.F. (2020). Creatine supplementation does not promote additional effects on inflammation and insulin resistance in older adults: A pilot randomized, double-blind, placebo-controlled trial. Clin. Nutr. ESPEN.

[135] Zamaraeva M., Sabirov R.Z., Maeno E., Ando-Akatsuka Y., Bessonova S.V., Okada Y. (2005). Cells die with increased cytosolic ATP during apoptosis: A bioluminescence study with intracellular luciferase. Cell Death Differ..

[136] Qu Y., Misaghi S., Newton K., Gilmour L.L., Louie S., Cupp J.E., Dubyak G., Hackos D., Dixit V.M. (2011). Pannexin-1 Is Required for ATP Release during Apoptosis but Not for Inflammasome Activation. J. Immunol..

[137] Scemes E., Spray D.C., Meda P. (2009). Connexins, pannexins, innexins: Novel roles of “hemi-channels”. Pflugers Archiv Eur. J. Physiol..

[138] Samuels S.E., Lipitz J.B., Wang J., Dahl G., Muller K.J. (2013). Arachidonic acid closes innexin/pannexin channels and thereby inhibits microglia cell movement to a nerve injury. Dev. Neurobiol..

[139] Kawamura H., Kawamura T., Kanda Y., Kobayashi T., Abo T. (2012). Extracellular ATP-stimulated macrophages produce macrophage inflammatory protein-2 which is important for neutrophil migration. Immunology.

[140] Zha Q.-B., Wei H.-X., Li C.-G., Liang Y.-D., Xu L.-H., Bai W.-J., Pan H., He X.-H., Ouyang D.-Y. (2016). ATP-Induced Inflammasome Activation and Pyroptosis Is Regulated by AMP-Activated Protein Kinase in Macrophages. Front. Immunol..

[141] Sakaki H., Tsukimoto M., Harada H., Moriyama Y., Kojima S. (2013). Autocrine Regulation of Macrophage Activation via Exocytosis of ATP and Activation of P2Y11 Receptor. PLoS ONE.

[142] Lee A.H., Ledderose C., Li X., Slubowski C.J., Sueyoshi K., Staudenmaier L., Bao Y., Zhang J., Junger W.G. (2018). Adenosine Triphosphate Release is Required for Toll-Like Receptor-Induced Monocyte/Macrophage Activation, Inflammasome Signaling, Interleukin-1β Production, and the Host Immune Response to Infection. Crit. Care Med..

[143] McArthur K., Whitehead L.W., Heddleston J.M., Li L., Padman B.S., Oorschot V., Geoghegan N.D., Chappaz S., Davidson S., Chin H.S. (2018). BAK/BAX macropores facilitate mitochondrial herniation and mtDNA efflux during apoptosis. Science.

[144] Hauser P., Wang S., Didenko V.V., Kalyuzhny A. (2017). Apoptotic Bodies: Selective Detection in Extracellular Vesicles. Signal Transduction Immunohistochemistry.

[145] Minton K. (2016). Anti-inflammatory effect of mitophagy. Nat. Rev. Immunol..

[146] Harris J., Deen N., Zamani S., Hasnat A. (2018). Mitophagy and the release of inflammatory cytokines. Mitochondrion.

[147] Bahat A., MacVicar T., Langer T. (2021). Metabolism and Innate Immunity Meet at the Mitochondria. Front. Cell Dev. Biol..

[148] Dhir A., Dhir S., Borowski L., Jimenez L., Teitell M., Rötig A., Crow Y.J., Rice G.I., Duffy D., Tamby C. (2018). Mitochondrial double-stranded RNA triggers antiviral signalling in humans. Nature.

[149] Jiang L., Paone S., Caruso S., Atkin-Smith G.K., Phan T.K., Hulett M., Poon I.K.H. (2017). Determining the contents and cell origins of apoptotic bodies by flow cytometry. Sci. Rep..

[150] Kalluri R., LeBleu V.S. (2016). Discovery of Double-Stranded Genomic DNA in Circulating Exosomes. Cold Spring Harb. Symp. Quant. Biol..

[151] Munoz L., Lauber K., Schiller M., Manfredi A.A., Herrmann M. (2010). The role of defective clearance of apoptotic cells in systemic autoimmunity. Nat. Rev. Rheumatol..

[152] Gupta S., Kaplan M.J. (2021). Bite of the wolf: Innate immune responses propagate autoimmunity in lupus. J. Clin. Investig..

[153] Sule S., Rosen A., Petri M., Akhter E., Andrade F. (2011). Abnormal Production of Pro- and Anti-Inflammatory Cytokines by Lupus Monocytes in Response to Apoptotic Cells. PLoS ONE.

[154] Li T., Chen Z.J. (2018). The cGAS–cGAMP–STING pathway connects DNA damage to inflammation, senescence, and cancer. J. Exp. Med..

[155] Röszer T. (2020). M2 Macrophages in the Metabolic Organs and in the Neuroendocrine System. The M2, Macrophage.

[156] Chobot A., Górowska-Kowolik K., Sokołowska M., Jarosz-Chobot P. (2018). Obesity and diabetes-Not only a simple link between two epidemics. Diabetes/Metab. Res. Rev..

[157] Sun K., Gao Z., Kolonin M.G. (2018). Transient inflammatory signaling promotes beige adipogenesis. Sci. Signal..

[158] Geserick M., Vogel M., Gausche R., Lipek T., Spielau U., Keller E., Pfäffle R., Kiess W., Körner A. (2018). Acceleration of BMI in Early Childhood and Risk of Sustained Obesity. N. Engl. J. Med..

